# Perceptions of Oral Nicotine Pouches on Reddit: Observational Study

**DOI:** 10.2196/37071

**Published:** 2022-07-15

**Authors:** Yihan Shao, Jonathan Zou, Zidian Xie, Rachel Grana Mayne, Deborah J Ossip, Irfan Rahman, Scott McIntosh, Dongmei Li

**Affiliations:** 1 Goergen Institute for Data Science University of Rochester Rochester, NY United States; 2 Department of Clinical & Translational Research University of Rochester Medical Center Rochester, NY United States; 3 Tobacco Control Research Branch National Cancer Institute Bethesda, MD United States; 4 Department of Public Health Sciences University of Rochester Medical Center Rochester, NY United States; 5 Department of Environmental Medicine University of Rochester Medical Center Rochester, NY United States

**Keywords:** oral nicotine pouches, Reddit, perception, nicotine, social media, sentiment, public opinion, user experience, attitude, content analysis, tobacco, smoking, cessation, quit, smoker, information seeking, information sharing, vaping

## Abstract

**Background:**

Oral nicotine pouches are a new form of tobacco-free nicotine products launched in recent years with a variety of flavors.

**Objective:**

This study aims to examine the public perceptions and discussions of oral nicotine pouches on Reddit, a popular social media platform for sharing user experiences.

**Methods:**

Between February 15, 2019, and February 12, 2021, a total of 2410 Reddit posts related to oral nicotine pouches were obtained over a 2-year period. After the removal of unrelated or commercial posts, 653 Reddit posts related to oral nicotine pouches remained. Topics and sentiments related to oral nicotine pouches on Reddit were hand coded.

**Results:**

The number of Reddit posts related to oral nicotine pouches increased during the study period. Content analysis showed that the most popular topic was “sharing product information and user experience” (366/653, 56%), in which sharing oral nicotine pouch products and user experiences were dominant. The next popular topic was “asking product-related questions” (product properties and product recommendations; 115/653, 17.6%), followed by “quitting nicotine products” such as vaping or smoking through use of oral nicotine pouches or quitting the oral nicotine pouches themselves (83/653, 12.7%) and “discussing oral nicotine pouch–related health” symptoms or concerns related to oral nicotine pouches (74/653, 11.3%). The least popular topic was “legality and permissions” related to oral nicotine pouches (15/653, 2.3%). In addition, a greater number of Reddit posts described positive attitudes compared to negative attitudes toward oral nicotine pouches (354/653, 54.2% vs 101/653, 15.5%; *P*<.001).

**Conclusions:**

Reddit posts overall had a positive attitude toward oral nicotine pouches and users were actively sharing product and user experiences. Our study provides the first insight on up-to-date oral nicotine pouch discussions on social media.

## Introduction

Oral nicotine pouches are popular products launched by tobacco manufacturers over the past 3 years [1]. In appearance, oral nicotine pouches are similar to snus, a smokeless tobacco product originated in Sweden. Instead of containing the tobacco leaf found in snus, they are composed of white nicotine-containing powder [2]. The properties of being spit-free and tobacco-free may make oral nicotine pouches attractive alternatives to smokeless tobacco products. A cross-sectional survey study found that almost all oral nicotine pouch users were either former or current tobacco users (96.3%) [3]. The market for oral nicotine pouches is rapidly growing, indicated by the sales volume of the global market, which is estimated at US $2.33 billion in 2020 and expected to reach US $21.84 billion by the end of 2027 [4]. Among a great variety of brands in the United States, ZYN, a Swedish Match product, by far occupied the largest national market share (nearly two-thirds), and shipments of ZYN in the United States have increased from 114.1 million cans in 2020 to 173.9 million cans in 2021 [5].

To date, several studies have investigated the chemical components of oral nicotine pouches. For example, a recent study examined the moisture content, pH, and nicotine content of 37 oral nicotine pouch brands [6]. They found a similar pH across products and nicotine content comparable to, or greater than, smokeless tobacco pouch products, suggesting a similar addiction potential as those products. Due to the high nicotine content, the authors expressed concern over appeal to, and use by, young people and that oral nicotine pouches may be a gateway to more harmful tobacco products. The various flavors contained in the oral nicotine pouches are another rising concern as flavors are one of the major reasons that youth are attracted to and use electronic nicotine delivery system products with high nicotine content [7-9]. Nevertheless, another study estimated the toxicant levels of oral nicotine pouches and concluded that users are likely to be exposed to lower levels of toxic compounds than with Swedish snus [10]. Given that Swedish snus has been shown to have significantly fewer health risks than cigarette smoking, these results indicate that nicotine pouch users may be at lower risk of adverse health effects than users of Swedish snus or cigarettes [10]. Potential health effects of oral nicotine pouches await further investigation. Although there have been studies about the chemical characteristics of oral nicotine pouches [6,10,11], few studies have investigated public perceptions of these products, the importance of which is highlighted further by the substantial and increasing use of oral nicotine pouches.

The universality of social media makes it a suitable platform to collect information on public attitudes and perceptions of new emerging trends. Reddit is a popular social media outlet that is differentiated from other social media platforms by its capability for users to form niche groups called “subreddits.” Each subreddit has a common topic and users within the subreddit can express their opinions and personal experiences. According to *The Wall Street Journal*, there are over 52 million daily active users of Reddit [12]. The posts on Reddit tend to have higher character limits, allowing users to provide more information on opinions and experiences, as well as have meaningful discussions with other users that have similar interests. Thus, Reddit posts on oral nicotine pouches contain more information on user opinions and product use experiences than posts on other social media platforms. A number of previous studies have used Reddit to examine user perceptions of other tobacco products, for example, public perceptions of different e-cigarettes on Reddit [13,14].

In this study, we aimed to examine the public perceptions of, and popular topics related to, oral nicotine pouches on Reddit during the past 2 years, when the sales of oral nicotine pouches significantly increased in the United States. First, to understand what is on market, we identified the brands and flavors of oral nicotine pouch products available online. Through manually coding Reddit posts for sentiments and topics, we examined public attitudes toward oral nicotine pouches and identified top topics related to oral nicotine pouches. Classifications of subtopics and analysis of sentiments within each topic were further conducted to obtain more details. The findings provide important information on this emerging nicotine product, which could be helpful for understanding perception, use, and future regulation of oral nicotine pouches.

## Methods

### Online Collection of Oral Nicotine Pouches

Coders (YS and JZ) searched online stores in February 2021 to identify oral nicotine pouch products and brands on the market during the Reddit data collection period and to inform the hand coding of the Reddit posts. Information about available oral nicotine pouch products, including brand names, product names, and flavors, was collected from online stores like “nicokik.com” and “snusdirect.com.” The nicotine strengths, when available, were also included.

### Data Collection and Preprocessing

Reddit data between February 15, 2019, and February 12, 2021, were downloaded from Reddit Archive [15] in March 2021. By keyword searching, 2410 posts containing keywords related to oral nicotine pouches and brand names, including “nicotine pouch,” “oral nicotine pouch,” “loop nicotine,” “lyft nicotine,” “ordic spirit pouch,” “zyn,” “velo,” “killapods,” “zonex,” “2one,” and “on!” were further extracted. Information including author, subreddit, created time, URL, and texts of the posts were included in our final data set.

To achieve higher accuracy of hand coding and defining the topics, we adopted an inductive approach [16]. We considered the method of content analysis, which is a research tool used to identify the presence of certain words, themes, or ideas in qualitative data [17]. We first randomly selected a sample of 350 posts (15% of the 2410 total posts), which was used to develop our initial codebook. Based on whether content was primarily focused on oral nicotine pouches or not, posts were first classified into related posts or unrelated posts. Unrelated posts were Reddit posts that did not mention oral nicotine pouches. Then, among related posts, we further classified them into commercial or noncommercial posts. Commercial posts were those directly posted by oral nicotine pouch sellers on Reddit, which contained advertisements for a specific product in text or had the URL directly linked to online stores of oral nicotine pouches. We focused our analysis on the related and noncommercial posts. Among the 350 posts, 233 of them were unrelated or commercial, and 117 posts were related and noncommercial. By applying the same rules to all posts, in total, 27.1% (653/2410) posts were related and noncommercial, 2.3% (56/2410) posts were commercial, and 70.6% (1701/2410) posts were unrelated (Multimedia Appendix 1).

### Topic Analysis

By examining the content of the 117 related and noncommercial posts, two authors (YS and JZ) individually grouped those with similar content and summarized them by a generalizable phrase such as “Sharing product information and user experience.” Within each general topic, we further summarized analogous posts by a more specific topic (subtopic) like “user experiences.” After hand coding 117 sampled posts separately, we compared and discussed our codebook definition from two individual coders, and the final decision on how to categorize topics was made by a group of 4 authors (YS, JZ, ZX, and DL) to form a unified classification standard. The initial codebook was used as the reference for the remaining 2060 posts, which were independently coded by 2 authors (YS and JZ). Any coding difference between the 2 coders was resolved through discussion among all 4 coders. Moreover, further modifications to the codebook were made in the process of hand coding the remaining 2060 posts with intensive discussions among the group of 4 coders. As a result, Reddit posts related to oral nicotine pouches were categorized into 5 topics: “sharing product information and user experience,” “asking oral nicotine pouch–related questions,” “quitting nicotine products,” “discussing oral nicotine pouch–related health,” and “legality/permissions” (Table 1). Each post was only assigned one topic or subtopic.

**Table 1:** Topics and subtopics of Reddit posts on oral nicotine pouches.

Topics and subtopics		Description
**Sharing product information and user experience**		
	Products	Posts where users were sharing about specific oral nicotine pouch products.
	User experiences	Posts where users were sharing the experience of how they were using oral nicotine pouches and how they felt about them when they were using the products.
	Opinions	Posts where users were sharing subjective points of view about the products.
	Information	Posts where users were sharing information that they obtained elsewhere.
	Others	Posts about other things like sharing homemade oral nicotine product recipes.
**Asking oral nicotine pouch–related questions**		
	Characteristics of oral nicotine pouches	Posts about the properties of oral nicotine pouches such as nicotine strength, flavors, and packaging.
	Product recommendation	Posts that were asking for recommendations of oral nicotine pouch products.
	Order or purchase information	Posts asking for information about the online and offline purchase of oral nicotine pouches.
	Others	Posts asking uncommon questions, like asking for opinions about the future of the oral nicotine pouch market.
**Quitting nicotine products**		
	Using oral nicotine pouches to quit vaping	Posts by users who were discussing intentions to use, attempts to use, or successful use of certain oral nicotine pouch products to quit vaping
	Using oral nicotine pouches to quit smoking	Posts by users who were discussing intentions to use, attempts to use, or successful use of certain oral nicotine pouch products to quit smoking
	Quitting oral nicotine pouches	Users of the posts intended to, were attempting to, or have successfully quit oral nicotine pouches
	Others	Posts that shared experience using oral nicotine pouches to quit other nicotine-related products like tobacco and nicotine itself
**Discussing oral nicotine pouch–related health**		
	Health symptoms	Posts discussing having negative health symptoms after the consumption of oral nicotine pouches, like gum bleeding and sickness
	Health concerns	Posts that expressed health concerns with oral nicotine pouches. Users of those posts expressed worries about the potential side effects of oral nicotine pouches, including the symptoms above and addiction to nicotine.
Legality and permissions		Posts that were discussing the regulatory policies and legal issues related to oral nicotine pouches

### Sentiment Analysis

In addition to analyzing the major topics discussed in each post, we performed sentiment analysis with the standard “positive,” “neutral,” and “negative” categories [18-22]. Example Reddit posts in each topic and subtopic category with different sentiments are shown in Multimedia Appendix 2. If the post expressed an overall positive attitude toward one of the oral nicotine pouch products or the oral nicotine pouches as a whole, we considered it as positive. For example, posts where users shared enjoyable user experiences with one of the oral nicotine pouch products or found oral nicotine pouches helpful for quitting other tobacco products were considered positive. On the other hand, if the post expressed an overall negative attitude, we considered it negative. For example, posts discussing having adverse health effects after using oral nicotine pouches or being pessimistic about the products’ future appeal were categorized as negative. Finally, if the post expressed neither an overall positive nor negative attitude, we labeled it neutral.

All the posts were double-coded. By focusing on 653 related and noncommercial posts, we calculated the agreement rates between topics and sentiments to examine the overall accuracy of classifications. The raw agreement rate (number of agreed posts/total posts) is 86.23% (563/653) for topics and 82.20% (537/653) for sentiments. The Cohen κ is 0.7526 for topics and 0.7102 for sentiments.

### Ethics Approval

The study has been reviewed and approved by the Office for Human Subject Protection Research Subjects Review Board (RSRB) at the University of Rochester (Study ID: STUDY00006570).

## Results

### Longitudinal Trend of Number of Reddit Posts

**Figure 1:**
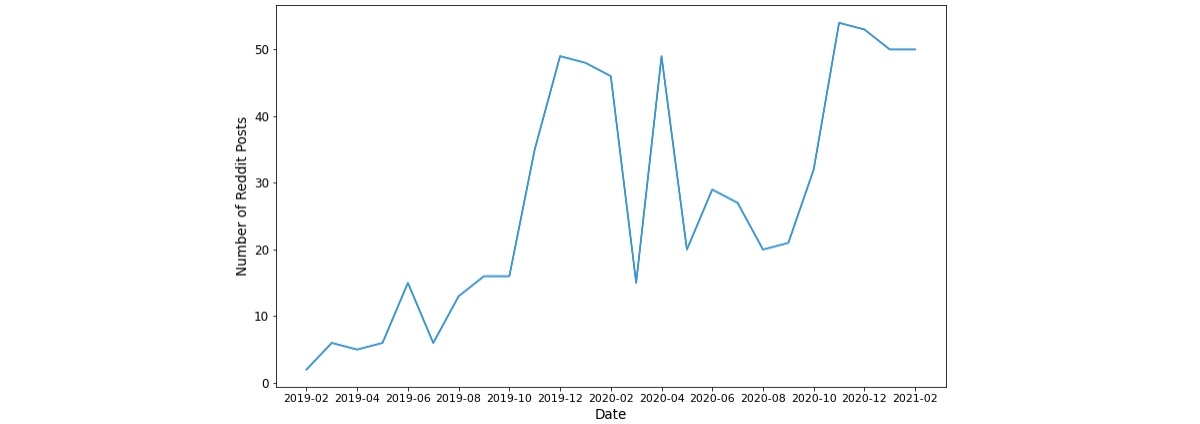
Longitudinal trend of Reddit posts related to oral nicotine pouches.

In total, coders (YS and JZ) identified 20 oral nicotine pouch brands and 30 different oral nicotine products available on online stores during the study period (Multimedia Appendix 3). To examine the trend in discussions about oral nicotine pouches on Reddit, we plotted the total number of posts per month from February 2019 to February 2021. As shown in Figure 1, there has been a clear upward trend in the number of posts related to oral nicotine pouches, with the most significant rise occurring from February 2019 (2 posts) to December 2019 (49 posts) and August 2020 (20 posts) to November 2020 (54 posts). Although the increase was steady in the two time periods mentioned above, very large fluctuations appeared between December 2019 and August 2020. Within that period, the number of posts peaked in April 2020.

### Topics and Subtopics

To understand the major topics discussed related to oral nicotine pouches on Reddit, we first divided posts into 5 general categories. Over half of the posts (366/653, 56%) were related to the topic “sharing product information and user experience.” The second most popular topic (115/653, 17.6%) was “asking oral nicotine pouch–related questions,” followed by “quitting nicotine products” (83/653, 12.7%) and “discussing oral nicotine pouch–related health” (74/653, 11.3%). Lastly, 2.3% (15/653) of posts reviewed the topic “legality and permissions.”

Among the 5 topics, 4 were further divided into subtopics. Under the topic “sharing product information and user experience,” users were more inclined to share “products” (140/366, 38.3%). Sharing “user experiences” accounted for the second-largest proportion (109/366, 29.8%), followed by sharing “opinions” (60/366, 18.6%). Sharing “information” and sharing “others” accounted for 10.7% (39/366) and 2.7% (10/366), respectively. For the topic “asking oral nicotine pouch–related questions,” almost half of the posts (57/115, 49.6%) were asking about “characteristics” of oral nicotine pouches. Approximately one-quarter (27/115, 23.5%) were asking for “recommendations.” Asking about “others” and asking for “order or purchase information” comprised 17.4% (20/115) and 9.6% (11/115), respectively. For the topic “quitting nicotine products,” over half of the discussions (42/83, 50.6%) were about “using oral nicotine pouches to quit vaping.” The remaining 3 subtopics were “using oral nicotine pouches to quit smoking” (15/83, 18.1%), quitting “others” (14/83, 16.9%), and “quitting oral nicotine pouches” (12/83, 14.5%). The fourth significant topic was “discussing oral nicotine pouch–related health.” When discussing health, the majority of posts (60/74, 81.1%) were talking about “health symptoms” related to oral nicotine pouches, and the rest talked about either “health concerns” (13/74, 17.6%) about oral nicotine pouches or “others” (1/74, 1.4%).

### Sentiments

Sentiment analysis of the Reddit posts determined that over half of the posts (354/653, 54.2%) expressed positive attitudes toward oral nicotine pouches (Table 2). Approximately 30% of the posts (198/653, 30.3%) showed neutral attitudes, while 15.5% (101/653) conveyed negative attitudes toward oral nicotine pouches. The proportion of positive posts was significantly higher than that of negative posts (*P* value of the two-proportion *z* test was <.001). With respect to each topic, “quitting” contained the most significant proportion of positive posts (57/83, 68.7%). “Product information/user experience” was also dominated by positive posts (233/366, 63.7%). On the other hand, the topic “health” had the highest proportion of negative posts (54/74, 73%) within all 5 topics. “Legality and permissions” had only 13.3% (2/15) negative posts. In the case of neutral posts, the topics “product-related questions” and “legality and permissions” contained the highest proportion (62/115, 53.9% and 8/15, 53.3%) of posts.

**Table 2:** Sentiments toward oral nicotine pouches in Reddit posts.

Topic	Positive, n/N (%)	Neutral, n/N (%)	Negative, n/N (%)
Sharing product information and user experience	233/366 (63.7)	99/366 (27)	34/366 (9.3)
Product-related questions	48/115 (41.7)	62/115 (53.9)	5/115 (4.4)
Quitting nicotine products	57/83 (68.7)	20/83 (24.1)	6/83 (7.2)
Discussing oral nicotine pouch–related health	11/74 (14.9)	9/74 (12.1)	54/74 (73)
Legality and permissions	5/15 (33.3)	8/15 (53.3)	2/15 (13.4)

## Discussion

### Principal Findings

This study explored user perceptions of oral nicotine pouches on Reddit by performing topic and sentiment analysis on 653 Reddit posts related to oral nicotine pouches collected over 2 years. We observed an overall increasing trend in the number of posts during the study period. Major topics about oral nicotine pouches include sharing preferred products, user experiences, or opinions of oral nicotine pouches; quitting oral nicotine pouches or using oral nicotine pouches to quit other tobacco products; and adverse health effects. Over half of the posts showed a positive attitude toward oral nicotine pouches. Although the majority of product information/user experience and quitting-related posts indicated positive sentiments similar to the overall sentiment, health-related posts contained the most significant proportion of negative posts.

### Comparison With Prior Work

It is interesting to note that the overall increase in the number of Reddit posts is consistent with the remarkable growth trend of sales of oral nicotine pouches during a similar time period. Compared to the sales of total other tobacco products in convenience stores in the United States for the 24 weeks ending on May 30 in 2020, which had a growth of 7%, sales of oral nicotine pouches grew a staggering 498% [23]. With regard to the striking fluctuations between February 2020 and August 2020, a potential and critical factor could be the global COVID-19 pandemic. The outbreak of COVID-19 constrained the tobacco product market in 2020 due to supply chains being disrupted by trade restrictions [24]. In addition, consumption and demand also declined because of lockdowns imposed by governments. It is therefore likely that the negative impact of the COVID-19 pandemic on the market and the public’s distraction by the pandemic contributed to the fluctuating interest in oral nicotine pouches reflected in the sudden rise and fall of the number of posts on the public subreddits included in our study.

Several factors could be responsible for the large proportion of posts discussing topics related to product information/user experience information of oral nicotine pouches. One factor could be that oral nicotine pouches are novel products, generating curiosity. Another factor could be the large variety of oral nicotine pouches brands and flavors, which further engenders curiosity, interest, and willingness to share their preferences and user experiences with others. Moreover, compared to other social media platforms like Instagram or Twitter, Reddit has a particular feature called a “subreddit,” where all users in the same subreddit are interested in the same topic and are only allowed to discuss related subjects [25]. For example, posts related to oral nicotine pouches were largely collected under subreddit like “NicotinePouches” and “zyn.” These oral nicotine pouch–related subreddits facilitated the discussions on oral nicotine pouches, which helped us understand the attitude and topics related to oral nicotine pouches through these commonly used platforms. However, due to the recent emergence of oral nicotine pouches, the subreddits “Snus” and “Nicotine” also contained relevant posts. This topic identity would create a more familiar atmosphere for users with the same interest, thus facilitating more active and positive sharing.

In this study, the overall sentiment toward oral nicotine pouches was positive, and most quitting-related posts were positive as well, which is consistent with Andersson and Lundqvist’s [1] finding that attitudes were more positive toward oral nicotine pouches than tobacco-containing snus [26]. The prevalence of positive posts relating to quitting smoking and vaping by switching to oral nicotine pouches might be due to misperceptions of the oral nicotine pouch products. Content analysis of quitting-related posts indicated that users (who had concerns about harmful effects of tobacco products such as cigarettes or e-cigarettes) considered the oral nicotine pouches as a “safer option” to help them quit vaping, as oral nicotine pouches contain sufficient nicotine content and provide a similar “buzz” as cigarettes or e-cigarettes. Some users may perceive the oral nicotine pouch products as useful for smoking cessation as a less hazardous form of nicotine, similar to nicotine replacement medication. Additionally, Struik and Yang [27] conducted a content analysis of a quit vaping community on Reddit and discovered that among 175 posts that mentioned methods of quitting, 13 users reported using nicotine pouches to help them quit e-cigarettes. In addition, those perceptions may be influenced by recent events in the oral nicotine product marketplace. In 2009, Reynolds American Inc (then the second-largest tobacco company in the United States) acquired Niconovum AB and in 2012 began test-marketing their nicotine replacement therapy products in the United States (Zonnic pouches and Zonnic mini lozenges) after obtaining approval from the US Food and Drug Administration (FDA) Center for Drug Research and Evaluation. However, in 2019, despite a national release and marketing in major convenience store chains, the company pulled the products from the US market for undisclosed reasons [28]. Currently, British American Tobacco, which owns Reynolds American and its subsidiaries [29,30], markets oral nicotine products (Velo, a nicotine pouch, and Revel, a nicotine lozenge) as commercial tobacco products [31], and has submitted a Premarket Tobacco Product Application to the FDA Center for Tobacco Products for the Velo product [32]. The proliferation of these products in the marketplace and the former availability of the Zonnic product as an FDA-approved cessation aid might engender confusion that using oral nicotine pouches can help with tobacco cessation [33,34]. In addition, the marketing of oral nicotine pouches as “tobacco-free” might convey to users that oral nicotine pouches are low-risk products, although there is a lack of evidence and there is no FDA authorization for those products as a modified risk tobacco product [35]. These findings broadly support the discovery of quitting-related posts of oral nicotine pouches in our study, as well as the unanticipated conclusion that the majority of these posts have positive sentiments.

Given the relatively short amount of time that oral nicotine pouch products have been on the market, the short- and long-term health effects of oral nicotine pouches use are still unknown and need further investigation. Our investigation of Reddit posts related to oral nicotine pouches identified some health symptoms mentioned by the users such as gum problems, which provided indications of what users may be experiencing and of potential health harms to be investigated. The effects of oral nicotine products on periodontal health and the upper airway of the lung are not known. There is a possibly that these products will cause oral submucous fibrosis [36]. Recent studies indicate that oral nicotine pouches are likely to expose users to lower levels of toxic components compared with snus and other tobacco products [10], though they are still addictive and could be damaging to human health [6]. Although health effects, especially long-term effects, of oral nicotine pouches remain to be determined, we showed in this study that there was an increasing trend of mentioning oral nicotine pouches, and that over half of related Reddit posts had positive attitudes toward oral nicotine pouches. Therefore, potential regulatory policy might be considered to prevent possible health concerns related to oral nicotine pouches.

### Limitations

The generalizability of these results is subject to several limitations. First, the paucity of noncommercial posts limited the scope of this study. Due to the fact that oral nicotine pouches are emerging new tobacco products, only 653 posts were included in our analysis during our study period from February 15, 2019, to February 12, 2021.

Thus far, there have been limited analyses of social media data on oral nicotine pouches. Our research provides a first and up-to-date insight into the current public perception and popular topics regarding oral nicotine pouches discussed on Reddit. Although this is a start, further research is needed due to the substantial growth of the oral nicotine pouch market. Our study only discusses the broad health symptoms mentioned in the Reddit posts. Whether those health symptoms are related to use of oral nicotine pouches and the health effects of oral nicotine pouches need to be investigated in future human studies.

### Conclusions

Our research provides a first insight into the current public perceptions on Reddit about oral nicotine pouches, which provide valuable information for regulators and policy makers. The trending topics summarized in our study were reflections of what the public was interested in regarding oral nicotine pouches. Our results only provided very preliminary information about the potential health effects related to oral nicotine pouches. Future studies are warranted to evaluate the health effects of oral nicotine pouches for regulation purposes.

## Appendix

Multimedia Appendix 1 Flowchart of data collection and preprocessing.

Multimedia Appendix 2 Example Reddit posts in each topic and subtopic category with different sentiments.

Multimedia Appendix 3 Online collection of oral nicotine pouch brands, products, and flavors.

## Abbreviations

FDA: Food and Drug Administration

## Data Availability Statement

Data are available upon reasonable request.
