# Supplementary figures and images for: Perceptions of Oral Nicotine Pouches on Reddit: Observational Study
Source: J Med Internet Res. 2022 Jul 15;24(7):e37071. doi: 10.2196/37071 (PMC9338421; doi:10.2196/37071)

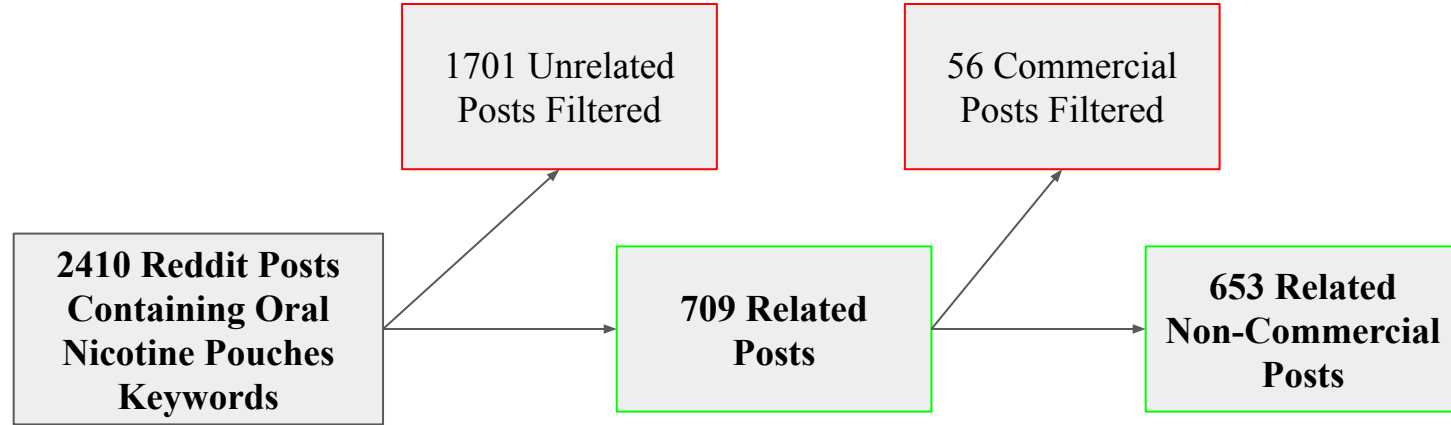

Supplement: Multimedia Appendix 1 [file jmir_v24i7e37071_app1.pdf]
