# Supplementary material for: Perceptions of Oral Nicotine Pouches on Reddit: Observational Study
Source: J Med Internet Res. 2022 Jul 15;24(7):e37071. doi: 10.2196/37071 (PMC9338421; doi:10.2196/37071)
Supplement: Multimedia Appendix 3 [file jmir_v24i7e37071_app3.docx]

**Supplemental Table 1. Online Collection of Oral Nicotine Pouches Brands, Products, and Flavors**

| **Brand name** | **Product name** | **Flavor** |
| --- | --- | --- |
| Rogue | Rogue puches 5 pack (6mg, 3mg) | wintergreen, peppermint, Mango, honey lemon |
| ZYN | ZYN 15 nicotine pouches (3mg, 6mg) | cool mint, peppermint, wintergreen, spearmint, cinnamon, coffee, citrus, smooth, chill |
| On ! | On 20 nicotine pouches (1.5mg, 2mg, 3.5mg, 4mg, 8mg) | Wintergreen, mint, cinnamon, coffee, citrus, berry, original |
| Velo | Velo max nicotine pouches 7mg | Wintergreen, Dragon fruit, citrus burst, mint, black cherry, spearmint, cinnamon, |
|  | Velo nicotine pouches 4mg | Wintergreen, dragon fruit, citrus burst, spearmint, cinnamon, peppermint |
|  | nicotine pouches (2mg, 4mg) | Mint, citrus |
| FRĒ | FRĒ nicotine white pouches (9mg, 12mg) | Mint, Mocha, Lush, and Flavorless |
| Triumph | Triumph Chew Pouch Single Cans (Full 5mg, Half 3mg, Quarter 1.5mg, Zero Strength (No Nicotine)) | mint, wintergreen, cinnamon, classic, Vanilla Bourbon |
|  | Triumph Pouches 5 and 10 packs (Full 5mg, Half 3mg, Quarter 1.5mg, Zero Strength (No Nicotine)) | mint, wintergreen, cinnamon, classic, Vanilla Bourbon |
| 2One | Adult nicotine satisfaction (3mg, 6mg, 8mg) | Eucalyptus rush, glacier mint, mocha java, wintergreen, whiskey&cola, gin&tonic |
| Dryft | 20 nicotine pouches | Citrus, wintergreen, dragon fruit, spearmint, cinnamon |
| LYFT | Slim Nicotine Pouches | Ice cool mint, freeze, mint, winter-chill, tropic breeze |
|  | Mini Nicotine Pouches | Easy mint, royal purple, ruby berry, liquorice, blonde roast, Caribbean spirit, london flair, berry frost, blueberry, easy mint mellow, lime, melon, Nordic winter, strawberry bloom, urban vibe |
| Ace | Ace Nicotine Pouches | Eucalyptus, cool mint, citrus, extreme cool, liquorice, mint |
| Loop | LOOP Nicotine Pouches | Mint Mania, Jalapeño Lime, Sicily Spritz, salty ludicris, |
| Shiro | Shiro Nicotine Pouches | Cool mint, Virginia Classic, true north, sweet, pina colada, mojito, cuba libre |
| Swave | Swave Nicotine Pouches | Daiquiri, Tropic Spritz |
| Sirius | Sirius nicotine pouch | Cold classic, ice cold |
| YOYO | YOYO Nicotine Pouches | London lime strawberry, new York mint, Stockholm Licorice Mint, Havana Mojito |
| Faro | Faro Nicotine Pouches | Raspberry mint, frosted, apple mint, iceshock, arctic, Acid Lemonsplash, original |
| NICO | NICO Strong All White | Alpine mint, Glacier mint |
| Nordic Spirit | Nordic Spirit Intense All White Portion | Spearmint |
|  | Nordic Spirit Slim All White Portion | Berry Citrus, Elderflower, Mint |
|  | Nordic Spirit Intense Strong All White Portion | Spearmint |
|  | Nordic Spirit Mini All White | Elderflower, Mint |
|  | Nordic Spirit Strong Mini All White | mint |
| Skruf | Skruf Super White Super Slim | Frozen shot, fresh, Nordic, polar, solbaer, |
| White Fox | White Fox Full Charge All White Portion | mint |
|  | White Fox Slim All White Portion | mint, spearmint |
|  | White Fox Double Mint All White Portion | mint-menthol |


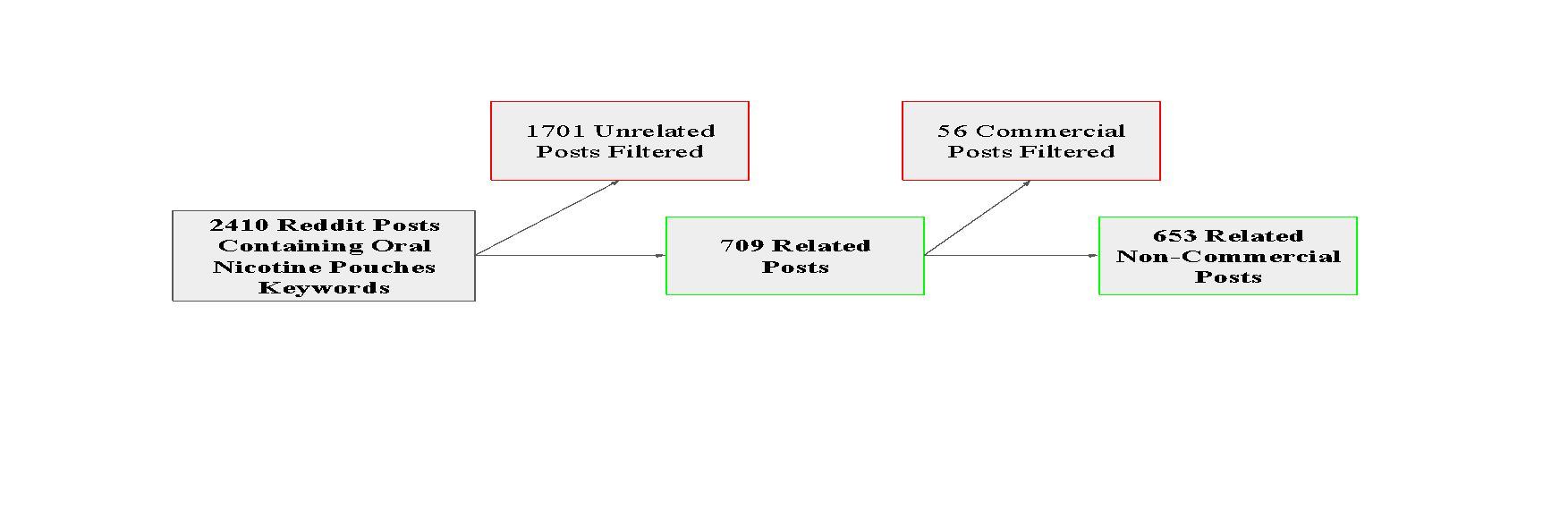


**Supplemental Figure 1. Flowchart of Data Collection and Preprocessing.**

**Supplemental Table 2. Example Reddit Posts in each Topic and Subtopic Category with Different Sentiments.**

| **Topics** | **Subtopics** | **Sentiments** | **Number of Posts** | **Reddit Post Examples with Different Sentiments** |
| --- | --- | --- | --- | --- |
| **Asking**  **Questions** | Seeking Nicotine Content Recommendation |  | 57 |  |
|  |  | Positive | 25 | I would love to know the nicotine content per pouch. I’ve been drinking and my math is shit. I think white tobacco is the answer to all my problems. I’m a huge spearmint fan. Opinions on flavor and strength? |
|  |  | Negative | 2 | I tried having a look and the lowest I could find was 2mg by Zyn. For some reason, I can tolerate nicotine gum well, but the 2mg of Zyn pouch caused my gums to sting and got me really dizzy. How can I adjust to this? |
|  |  | Neutral | 30 | Like what would be the nicotine pouch equivalent of a puff bar or juul pod? |
|  | Product Recommendation |  | 27 |  |
|  |  | Positive | 15 | Recently I tried zyn espressino and I must say it is pretty decent, especially as a desert pouch. The size is really nice too super slim, but strong in nicotine. Some zyn recommendations? Recently I became big fan of their products. |
|  |  | Negative | 2 | I’ve tried On! Nicotine pouches and they just seemed a bit too dry for me and ripped my gums to shreds. What are your guys/gals opinions on a good nicotine pouch that isn’t terribly dry and/or won’t tear up my mouth? |
|  |  | Neutral | 10 | Can anyone suggest an equivalent snus from the Lift X-Feeze portion that I can order in Canada ? Now that we cannot order nicotine pouches in Canada, I would need to find a similair product. Thanks ! |
|  | Order/Purchase Information |  | 11 |  |
|  |  | Positive | 4 | Hello everyone! I am Russian, living in France now. Just writing to ask if I can find any EU citizens who order snus or nicotine pouches. Share the experience about the stores and delivery. For me, Northerner com works perfect, ups delivery in 3 days, or sometimes not (with ups) 6-10 days. I did not have any issues with them! And I once ordered with Snusline (they can sell Siberia and other stuff like this). It took like 35 days to deliver and I felt really nervous! But I have it now under my lip and I am fine:) Please, tell me, which stores you use for Europe? |
|  |  | Negative | 0 | None |
|  |  | Neutral | 7 | Anybody has a good recommendation for ordering Fox Double Mint cheap to Germany ? I looked at all the recommendations in the other thread but delivery made it very pricey (about 40 Euros only delivery on odensnus) |
|  | Other |  | 20 |  |
|  |  | Positive | 4 | I put a Zyn pouch between my lip and gums to help generate saliva for a COVID test (and for the buzz of course) but it’s now occurring to me, could the nicotine infused Saliva give the result of a false negative? |
|  |  | Negative | 1 | Do you think the extremely high nicotine pouch market (50mg and up) will destroy any progress made with real Snus and normal strength pouches? I can see a crack down in the future personally. |
|  |  | Neutral | 15 | Does anybody else get a good sneeze from the first snus/nicotine pouch you put in after not having one for a bit? |
| **Sharing Experience** | Products |  | 140 |  |
|  |  | Positive | 119 | I like the Rogue pouches the most. Zyn are good too, but only 15 pouches per can. |
|  |  | Negative | 3 | I tried the zyn. Boooooo |
|  |  | Neutral | 18 | Zyn nic pouches I believe are slim. On! Nicotine pouches are mini. |
|  | User Experience |  | 109 |  |
|  |  | Positive | 66 | I personally preferred Zyns over vaping or smoking because it gave me a better head rush and was discreet so I could do it whenever I wanted. |
|  |  | Negative | 15 | I tried this and its complete shit. I'm a huge snus fan, but Velo doesnt fit into any of the categories we cover in this forum in my opinion. I dont understand this stuff because it has no flavor and I dont think I'm taking in any nicotine. |
|  |  | Neutral | 28 | Rn im using 50mg/g pablo nicotine pouches |
|  | Opinion |  | 68 |  |
|  |  | Positive | 22 | I like it. If I switched to nic pouches it would be in my full time rotation |
|  |  | Negative | 15 | General snus is certainly worth a try. Worlds better than Zyn. |
|  |  | Neutral | 31 | Honestly this is such a new product, anything you would get from ZYn would be speculation. Go see a doctor if you are concerned for your health, honestly report back the findings from your family doctor. We all should know by now how big companies operate don't trust us or other media follow what your doctor says and if you we'll post back here. I never had an issue but fuck that let us know once you see a doctor. |
|  | Information |  | 39 |  |
|  |  | Positive | 17 | I agree. It's the future of snus as well. Not to mention many people are here for harm reduction and I see nic pouches as a continuance of that. Just look at Swedish producers such as epok which switched entirely to tobacco free and now produce Lyft exclusively. |
|  |  | Negative | 1 | More of these dangerous high nicotine Russian products.  <https://www.interfax.ru/russia/686902> |
|  |  | Neutral | 21 | I never had your issues with Dryft but I will chime in to say Dryft is being rebranded as Velo by BAT in the US but it will not be the same as the current Velo in the US or the EU. At least from my understanding |
|  | Other |  | 10 |  |
|  |  | Positive | 9 | Homemade Moist Coffee Nicotine Pouch |
|  |  | Negative | 0 | None |
|  |  | Neutral | 1 | I'm a little scared for when I fly back to Canada now. I'm currently in the uk visiting family and I bought like 50 cans on nic pouches to bring back. Crossing my fingers my bag doesn't get checked then... |
| **Quitting** | Using Nicotine Pouches to Quit Vaping |  | 42 |  |
|  |  | Positive | 37 | I'm currently on day 3 of no vape and have been using zyn and dryft pouches. They work really well surprisingly. I get a good buzz on my first one of the day but nothing substantial after that but they do help a lot. I still want to vape at times but every time I get a craving I just pop one in and it subsides. |
|  |  | Negative | 0 | None |
|  |  | Neutral | 5 | What do you mean by "fluff" it? Is that something ur supposed to do with Zyn? I'm using it to quit vaping and I have no clue' |
|  | Using Nicotine Pouches to Quit Smoking |  | 15 |  |
|  |  | Positive | 9 | I used zyn while I was waiting on a snus order for about a week because i had just quit smoking and its the only alternative my local gas station had to offer. I think it was the 6mg wintergreen. No problems at all, kept me okay when i needed a fix. Now im on lundgrens, but zyn is A okay in my book |
|  |  | Negative | 1 | Hey fellas. I’m looking for alternatives to smoking (was not a heavy smoker, 5 smokes a day, winston reds) since my current landlord is super anal about the smell (even outdoors) and so far vaping has done jack shit for me. I tried ZYN earlier today but it made me feel sick at 6mg a pouch. I’d like to go for actual tobacco instead of nicotine salts but I’m not sure where to start. |
|  |  | Neutral | 5 | I'm a smoker and am trying to do so less, to eventually stop. I found these nicotine pouch things that are nicotine salt, and wanted to make sure that if using them that they wouldn't interfere with my meds I take for seizures, and to make sure there isn't any side effect to them that provokes seizures. The company that makes them is called On! |
|  | Quitting Nicotine Pouches |  | 12 |  |
|  |  | Positive | 3 | I used zyn for probably a week and then just bit the bullet and went off nic completely. I started with probably 3-4 pouches a day and tried to cut back to 1-2 before I just stopped altogether. If you can, just get rid of all of your nicotine before winter break, go home and deal with the withdrawals and beat addiction there. Much easier imo. |
|  |  | Negative | 4 | Zyn was as bad as vaping because each pouch had a shit ton of dose, and I would kill a pack a day. Here we are now day 2 no nicotine... withdrawals are killing me, I feel so mad at the world for no reason. I know I’ll feel better soon!! |
|  |  | Neutral | 5 | I developed pretty big nicotine addiction while being in the quarantine and I decided to end it. I had a big temptation to have just one nicotine pouch but I resisted! |
|  | Others |  | 14 |  |
|  |  | Positive | 8 | For me personally when I gave up on snus and nicotine I switched from snus to lyft and zyn. I loved the lyft lime and liquorice from lyft. With zyn my go to was apple mint and blood orange and ginger. Very nice taste and not to strong. |
|  |  | Negative | 1 | Zyn is not marketed nor designed as a cessation product, it is meant to keep you hooked. I stopped juuling when it became harder to get pods in MA and for the past five months I have been heavily addicted to Zyn. It is a good substitute to the Juul, not a good way to quit nicotine. |
|  |  | Neutral | 5 | I like this approach. Tapering is a valid approach. I've haven't had long cut in a month. Im using the precision dosing of 6 mg pouches to taper off of nicotine. I'm down to 1 to 3 pouches from 1 to 2 cans of Kodiak per day. I've laid out a calendar. I'll switch to 3 mg pouches Monday. |
| **Health** | Health Symptoms |  | 60 |  |
|  |  | Positive | 7 | I've been using the pouches since May after quitting vaping .. just quit the pouches a couple days ago. Rogue was any favorite and i chained them constantly . Just recently before deciding to quit , my gums felt the exact same way where i kept the pouches . super sensitive . |
|  |  | Negative | 47 | I'm in the same boat. 2 cans of ROGUE in the last 5 days has stained my teeth, middle bottom teeth are sore and countless layers of skin has come off of my inner lip. Gums have noticeably receded. I just googled if anyone else was having issues with ROGUE. |
|  |  | Neutral | 6 | good luck managing your tolerance. I haven't had any kind of buzz from nicotine in years. I switched to zyn and the like last year. Getting some mild gum recession on the upper gumline so I'm switching spots to let that heal. |
|  | Health Concerns |  | 13 |  |
|  |  | Positive | 3 | Zyn helped me not crave cigs. It also helped with some anxiety I'd get at work around my superiors. I enjoy it because it's conspicuous and doesn't bite as much as gum. I have a medical background, and I don't think it's completely risk free, but I think it's a heck of a lot safer than smoking and chewing. Nicotine causes your blood vessels to shrivel up temporarily, so I can see that being an issue if used chronically in terms of oral health |
|  |  | Negative | 7 | I was wondering this too. Over on r/snus they say nicotine pouches can cause gum recession and I'm a little paranoid now. |
|  |  | Neutral | 3 | The last couple months I started using nicotine pouches to quit smoking. It worked! However, I’ve felt that my gums are starting to erode a little bit. Having said that, I feel much better anyway, lungs feel great and throat, but I was wondering whether the nicotine pouches are better than tobacco snus. I’ve come to love the feeling of the pouch in my gum and was wondering if the effects of regular tobacco snus is too much worse for my gums. |
|  | Others |  | 1 |  |
|  |  | Positive | 1 | Then I use a 6mg Zyn nicotine sublingual pouch (start with 3mg). Within 10 minutes you'll have to go to the bathroom. I have to do this everyday because there's something wrong with me that's undiagnosed. Just sharing cause I wish I knew this earlier!! |
|  |  | Negative | 0 | None |
|  |  | Neutral | 0 | None |
| **Legality/**  **Permissions** |  |  | 15 |  |
|  |  | Positive | 5 | Does anyone know what the rules are during the LSAT-Flex for packing a pouch? Zyn helps me focus but I have to spit every couple minutes. Is this allowed or not? If not I suppose I could use the ones with lower nicotine and just swallow it but I'd rather not. |
|  |  | Negative | 2 | Due to a recent news report, we will no longer review any snus, nicotine pouch, or chew bag product from Russia. Also, due to their lack of standards on these products, and lack of quality control, as well as the insanely high nicotine strengths, we do not support or encourage use of these products. |
|  |  | Neutral | 8 | It doesn't have to do with legal restrictions, here in norway LYFT is literally band and illegal because it DOESN'T have tobacco (epok is legal tho since it has white tobacco) |
